# The Effect of Different Cleaning Protocols on Post Space: A SEM Study

**DOI:** 10.1155/2016/1907124

**Published:** 2016-09-28

**Authors:** Giuseppe Lo Giudice, Angelo Lizio, Roberto Lo Giudice, Antonio Centofanti, Giuseppina Rizzo, Michele Runci, Angela Alibrandi, Marco Cicciù

**Affiliations:** ^1^Medical Sciences and Odontostomatology Department, University of Messina, Messina, Italy; ^2^Department of Neurosciences, Reproductive and Odontostomatological Sciences, University of Naples Federico II, Naples, Italy; ^3^Department of Economics, University Messina, Messina, Italy; ^4^Human Pathology Department, University of Messina, Messina, Italy

## Abstract

*Aim*. Purpose of the present paper is to analyze the efficiency of different post-space irrigation protocols.* Methods*. 28 single rooted teeth were endodontically treated. After post-space preparation every sample was assigned to one of three experimental groups and to one control group. In each group different irrigation protocols were performed as follows: EDTA (Group A), 37% orthophosphoric acid (Group B), and EDTA + 37% orthophosphoric acid with ultrasounds activation (Group C). In the control group (Group D) the irrigate association was not activated by ultrasounds. Three zones (coronal, middle, and apical) of each sample were analyzed by using Scan Electron Microscopy (SEM) without any metallization procedures. The presence of smear layer on the canal surface was qualitatively evaluated by applying Serafino's score with values included between 0 and 2.* Results*. The results of the research showed how Group C recorded the better results (0.81 ± 0.72). Group A and Group B showed lower mean scores (1.06 ± 0.69 and 1.08 ± 0.77); Group D showed the lowest mean score of 1.30 ± 0.69. The SEM observation analysis demonstrated how the smear layer presence decreased in the crown-apical direction.* Conclusions*. The different post-space treatments statistically determine significant differences on the dentinal surfaces cleansing. The absence of ultrasonic activation lowers the cleansing efficacy of endocanalar irrigants, showing sensible differences among each post-space zone.

## 1. Introduction

During the last years the research in the restorative-prosthodontic field has led to the development to post-and-core metal free system for the post-endodontic treated teeth. The use of reinforced fiber posts has showed excellent performance in many studies due to its elastic modulus similar to teeth's dentinal substratum [1–13]. Usually, the post has been passively positioned insides the root canal. The retention is therefore guaranteed by the cementation system [1, 3, 6].

The weak point of the whole tooth-posts-restoration system may be identified in the adhesion between the substratum and the resinous cements [2, 14]. Many in vivo and in vitro studies have showed how the fiber post reconstruction failure is dependent on the bonding-dentin interface [10, 11, 15, 16]. To achieve the best adhesion the optimal interface preparation is related to the bonding agents [14]. The bonding mechanism exploits the dentinal tubules penetration by the resin and the collagen fiber exposition. By this way, the canal surface should be cleaned and conditioned to let the formation of the hybrid layer and resin tags [1, 3, 14, 17, 18].

Recently the dentin “surface conditioning” consisting in a treatment of partial or complete smear layer removal in order to preserve the smear plugs was proposed [19].

However, in the literature it is not clear how to modify the dentin post space before the cementations procedure [1].

The etching using the orthophosphoric acid 30% to 40% is the most common technique for the enamel conditioning and for the smear layer removal [2, 20–23]. Other authors suggest that other irrigants such as EDTA and sodium hypochlorite at different concentrations may be variously alternated among them [24, 25].

The introduction of ultrasonic technology in endodontics has led to proposing new irrigation protocols for cleaning the post space [3, 26]. However, the literature results are contradictory and low debated regarding the quality of endodontic and post-space cleaning with this activation system [3, 21, 26–28]. Serafino et al. have shown that the cleansing of the post space made with 17% EDTA and ultrasound, followed by etching, seems to be the most efficient method compared to using only orthophosphoric acid [21].

The purpose of this in vitro study is to evaluate the degree of the post-space dentin cleaning. The research evaluated different irrigation systems that combine the use of 17% EDTA and etching by orthophosphoric acid at 37% with or without ultrasonic activation.

## 2. Materials and Methods

28 sound monoradicular dental elements, extracted for periodontal reasons and without previous conservative, endodontic, and/or prosthetic treatment, were used.

The presence of a single channel was verified by digital radiographic evaluation in mesiodistal and buccolingual projections.

In order to prevent deterioration, each dental element has been preserved after the extraction in 1% thymol solution for a period not exceeding thirty days.

Before being subjected to endodontic treatment, the all elements were cut at the CEJ level using a truncated-conical shape diamond bur 0.16 medium.

The access cavity was then created; the patency of the root canal was confirmed by K-file # 10. To check the working length, 4x magnification binocular glasses were used (EyeMag Pro S Zeiss, Carl Zeiss S.p.A. Milan, Italy). It was possible to control that the endodontic instrument did not exceed the apical foramen [29–31].

The endodontic treatment was performed using the base sequence of the Protaper system (S1, S2, F1, and F2), alternating washes with NaOCl (3 mL to 5.25%) conveyed by a needle with a lateral opening (27 Gauges) and positioned at a distances between 1 and 2 mm from the predetermined working length for each sample.

After the cleaning and shaping phase, the channels were dried using sterile paper cones. Then the channels were filled with gutta-percha cones and zinc oxide and eugenol cement (Pulp Canal Sealer EWT Kerr) using the continuous wave of condensation technique (System B) for the downpacking and Obtura syringe for backfilling.

Each treatment was confirmed by using digital radiography.

The next phase consisted in the creation of two parallels to the channel grooves on the mesial and distal surface of the roots using a tungsten carbide disc to achieve a predetermined fracture plane of the sample. This phase was carried out after the root filling, to avoid possible contamination of channel with the debris produced by the milling dental element.

Then the gutta-percha was removed for the creating of the post space. Extending the preparation up to 4 mm from the working length to ensure apical seal in gutta-percha used largo cutters from # 1 to # 4.

The samples were divided into four groups of observation (A, B, C, and D).

Each group was subjected to a different system of irrigation and of dentin conditioning:

- Group A: irrigation with 17% EDTA for 15 seconds with ultrasonic activation.
- Group B: etching with phosphoric acid 37% liquid for 15 seconds with ultrasonic activation.
- Group C: irrigation with 17% EDTA for 15 seconds with ultrasonic activation and subsequent etching with phosphoric acid 37% liquid with ultrasonic activation.
- Group D (control group): irrigation with 17% EDTA for 15 seconds and subsequent etching with phosphoric acid liquid to 37% for 15 seconds.

The irrigating agents were conveyed in the post space with a side opening needle (27 Gauges); the ultrasonic activation was performed with endodontic files steel # 20 EMS inserted in Endochuck 120° mounted on an ultrasonic handpiece EMS Castellini. Washing was performed with demineralized H_2_O in the C and D groups samples between irrigation and etching. During the ultrasonic activation phase, specific attention was paid to ensure that the files worked passively in the channel.

At the end of each procedure the channels were irrigated with distilled water and then sterile paper cones were used for drying.

Once the samples were prepared the buccolingual direction fracture was performed for exploiting the previously performed incisions.

**Figure 1:**
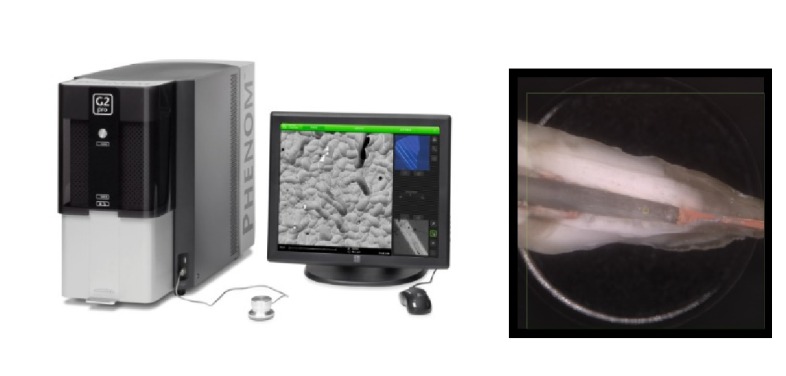
The SEM used and the low magnification image used for the observation zone determination.

The root canals surface was observed with a scanning electron microscope Phenom G2 pro (Phenom-World BV, Eindhoven, Netherland) (magnification range 20–45,000x, resolution 25 nm) (Figure 1). This microscope investigation let the observation of the sample in the absence of metallization procedures. The samples therefore have not undergone any fixations treatment (Figure 1).

The observations were conducted at 1.000x and 2.000x at the apical third, middle third, and the coronal third of the endocanalar root region. To achieve an adequate observation field, it was preferred to perform multiple observations in each third in order to obtain a continuous band of extended dentin in the mesiodistal direction.

In each band of observations there were three areas of 16.9*∗*10^2^ *μ*m^2^ randomly identified in which the evaluation was carried out of each group of samples, using the score according to Serafino et al. [21].

**Figure 2:**
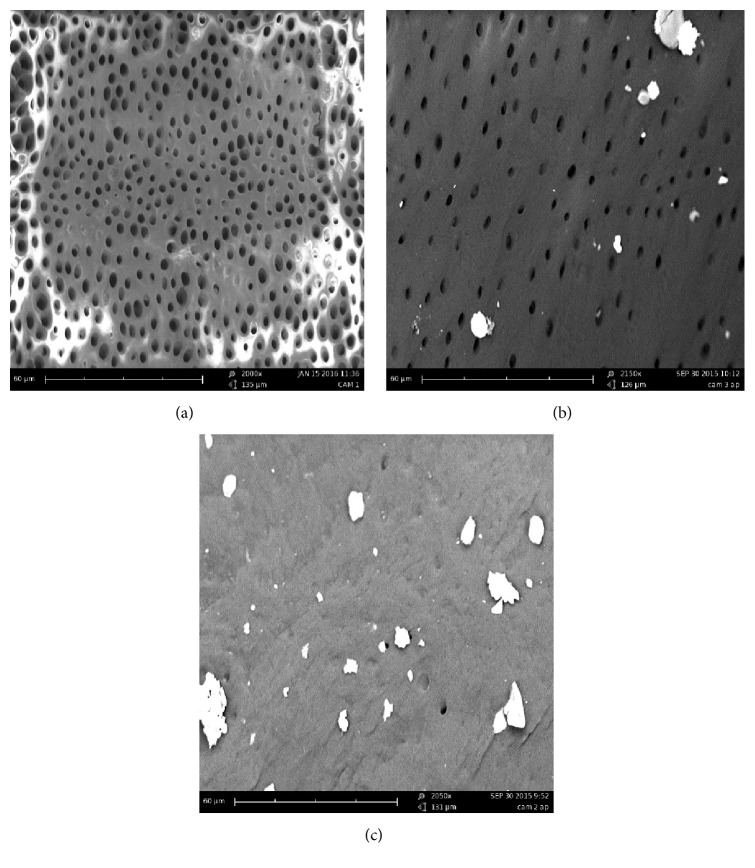
SEM observations. Scores examples: 0 (a), 1 (b), and 2 (c).

According to this evaluation method, the amount of debris is identified with a score between 0 and 2:

- Score 0: no debris, patency of dentinal tubules.
- Score 1: debris with a diameter smaller than 20 *μ*m and in limited quantities.
- Score 2: debris with a diameter greater than 20 *μ*m and in high numbers, impossible to display the entrance of dentinal tubules (Figure 2).

Two operators performed the score evaluation with a study conducted in double-blind. So the results could not be influenced by the conscious or unconscious expectation effects that would lead to invalidation of the results. The evaluation was followed by a statistical analysis of the results obtained on the control sample and the experimental groups, in order to highlight any significant differences.

Scores related to zones and groups were expressed as mean and standard deviation.

In order to perform comparisons among zones (coronal, middle, and apical), Kruskal Wallis test was performed for each group (A, B, C, and D); since the results were statistically significant, we performed pairwise comparisons between zones for each group, applying the Mann Whitney test; especially for these multiple comparisons, we applied Bonferroni's correction, through which the significance level *α* = 0.050 has to be divided by the number of the three possible pairwise comparisons, so the new “adjusted" significance level for this analysis is equal to 0.050/3 = 0.017.

In order to compare the scores among groups (A, B, C, and D), the same Kruskal Wallis test was performed for each zone (coronal, middle, and apical) and globally (in toto); the test results are significant and for this reason we performed pairwise comparisons between groups, applying Mann Whitney test; after Bonferroni's correction, the “adjusted" significance level for this analysis is equal to 0.050/6 = 0.008.

Statistical analysis was performed using SPSS 17.0 for Window package. *p* < 0.05 two sided was considered to be statistically significant.

## 3. Results

The results of our research show the best average score 0.81 ± 0.72 obtained in group C (37% phosphoric acid and EDTA 17% ultrasonically activated).

In Groups A and B, respectively, characterized by EDTA and phosphoric acid ultrasonic activation, lower average values were obtained than the control group (resp., 1.06 ± 0.69 and 1.08 ± 0.77) but higher than Group C.

**Figure 3:**
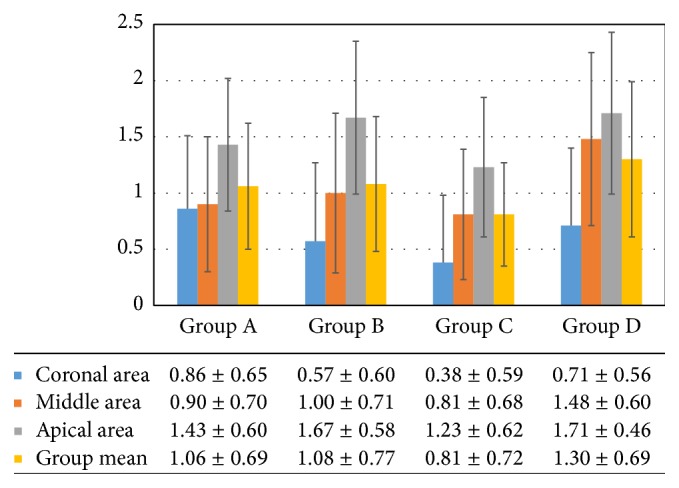
Groups and different areas average scores.

The control group (Group D) registered the lowest level of cleansing among the four groups, with an average score of 1.30 ± 0.69 (Figure 3).

The comparison, within each group of samples (A, B, C, and D) in the 3 observation areas proceeding in crown-apical direction showed how the score gradually increases. In Group A, the average values were 0.86 ± 0.65 in the coronal third, 0.90 ± 0.70 in the middle third, and 1.43 ± 1.58 in the apical third. In Group B the values were 0.58 ± 0.60 in the coronal third; 1.00 ± 0.71 in the middle third, and 1.67 ± 0.58 in the apical third. Group C showed average values of 0.38 ± 0.59 and then moves to 0.80 ± 0.68 and 1.23 ± 0.62. Group D (control) showed the values 0.71 ± 0.56 for coronal third, 1.48 ± 0.60 for middle third, and 1.71 ± 0.46 for the apical third (Figure 3).

The statistical evaluation showed significant differences between the 3 observational zones (coronal, middle, and apical) within each group of samples (A, B, C, and D). Group D has the highest degree of significance with a *p* value (*p* = 0.00001), following Group B with *p* = 0.0002, Group C with *p* = 0.00041, and Group A with *p* = 0.01203.

In pairwise comparison between the observation areas within the same group, a significant difference is evident in all groups between the coronal area and the apical area. Group A and Group B showed significant difference between the middle third and apical third (*p* = 0.016 e *p* = 0.002, resp.); in Group D (control), the difference was significant between the coronal third and middle third (*p* = 0.0003).

Comparing the four examined groups, a significant difference is shown between the middle third and apical third, respectively, with a *p* value equal to 0.012 and 0.027. The pairwise comparison between the different groups highlights a high statistical significance linked to the different score of the middle third cleansing in the comparison between A and D (control) groups (*p* = 0.007) and in the comparison between C and D (control) groups (*p* = 0.003); the differences described are highly significant, being lower than *p* = 0.008 after Bonferroni's correction. Regarding the coronal area, the comparison between A and C groups (*p* = 0.015) shows a tendency to significance, with the *p* value being superior to the corrected significance level itself; same considerations could be done for the apical area in comparison between B and C groups (*p* = 0.019), for the middle area in comparison between B and D groups (*p* = 0.028), and for the coronal and apical areas in comparison between C and D groups (*p* = 0.047 and *p* = 0.010, resp.).

In pairwise comparison between groups, considering all the zones, there are statistically significant differences between Groups C and D (*p* = to 0.0002).

## 4. Discussion

The data emerging from the research have shown that the different treatment of post-space results in a significant quality difference of the cleaning of the dentin surface.

Several irrigation protocols have been proposed, although studies in the literature are contradictory and not very numerous [3, 26].

Chemical agents, such as NaOCl, H_2_O_2_, EDTA, chlorhexidine digluconate, citric acid (10%, 20%, and 50%), orthophosphoric acid (H_3_PO_4_), and combinations of these, have been proposed for the removal of the smear layer [2, 17].

The experimental design of our research involved the use of 17% EDTA and 37% phosphoric acid in various combinations.

Demiryürek et al. have shown that different treatments for the removal of cement residues and smear layer from root canal surface affect the adhesion strength of a fiber post [2]. In particular, the presence of the smear layer potentially interferes with the polymerization of the resinous adhesive 11 materials [2, 8] for the production of free oxygen radical. The same AA have shown that the use of 17% EDTA followed by 5% NaOCl produces a complete removal of the smear layer by determining superficial erosions with full opening of the dentinal tubules [2].

Numerous other studies recorded the alternating irrigation with EDTA and NaOCl effectively responsible of the smear layer removal [24, 25, 32]. However, the use of 17% EDTA for 5 minutes alone could cause a severe erosion of the dentin root surface due to excessive demineralization [8, 13, 16, 25].

Saito et al. claim that reducing the irrigation time with EDTA below 1 minute can significantly decrease the smear layer removal [33].

Our research analyzed the effectiveness of the cleansing carried out with the activation of irrigating with ultrasound.

Gu et al. have reported that a 1-minute time EDTA irrigation can effectively remove the smear layer, while the NaOCl can remove the smear layer on a larger part of the dentin surface but the apical portion of the post space. However, the ultrasonic irrigant activation would not have a relevant effect [3].

Even Hülsmann et al. have reported that the additional use of ultrasound does not increase the cleansing ability of irrigating solutions [27].

In another study, Coniglio et al. highlighted the possibility of reaching an adequate level of cleaning due to the combination of ultrasonic tips and 17% EDTA [26]. This is in accordance with what is shown by Plotino et al., according to which the ultrasonic vibration would increase the effectiveness of irrigating solutions in removing debris [28].

Serafino et al. argue that the activation of ultrasonic irrigating solution followed by etching provides effective cleaning even on the apical third of the post space [21].

The differences between the irrigation protocols used in our research are highlighted by different amount and distribution of debris on the surface of the post space.

The statistical analysis shows significant differences between the 3 observational zones (coronal, middle, and apical) within each group of samples (A, B, C, and D). Taking into account the three levels of observation, the average score within each group shows a descending cleaning level towards the apical direction.

In particular, the D control group (irrigation with EDTA/orthophosphoric acid without ultrasonic activation) is characterized by a significant difference between the areas (lowest *p* value). This condition indicates the lack of ultrasonic activation and a consequent lack in cleansing of root canal surface moving in the apical direction.

Group B also shows a significant difference between the areas; the action of orthophosphoric acid with ultrasonic activation is not clearly effective to obtain a homogeneous cleaning.

Groups C and A, respectively, characterized by the activation of the orthophosphoric acid/EDTA and only EDTA showed higher *p* value, a sign that the EDTA activation would ensure a more uniform cleaning of the root canal surface. In an average comparison of the last few groups it is evident that the best value was found in Group C, which also shows the lowest absolute values.

The pairwise comparison between the observation areas, as part of the samples treated with the same protocol, showed a significant difference between the coronal area and the apical area in all groups, confirming that cleaning would become less effective moving apically.

In Group A and Group B there is a significant difference in the comparison between the middle third and apical third; it is therefore assumed that the action of the two individually activated irrigants is more likely to lead to a lower degree of cleansing when compared with Group C where the irrigant action tends to be more uniform. In Group D (control), the absence of activation determines a significant difference also between the coronal area and average.

In the comparison between groups there was a significant difference between the middle third and apical third; cleaning was more difficult moving apically, it is evident that the strengthen action of ultrasounds causes significant differences more in the middle zone (*p* value = 0.012) rather than the apical (*p* value = 0.027) of the post space.

Regarding the areas in paired groups, it is evident that comparing the middle zone of Group A versus D and the middle zone C versus D demonstrated the increased efficacy of activated EDTA.

Evaluating significance trends can highlight that the samples of Group C are generally more cleansed then those of Group A and Group B, but it should be emphasized especially that there is a tendency to statistical significance in the comparison between Groups A and D, to further confirm the effectiveness of EDTA ultrasonic activation.

## 5. Conclusions

The data analysis shows how the different post-space irrigation protocols correspond to different types of cleaning.

However, the amount of debris remaining tends to increase from coronal to apical area.

The protocols that used ultrasound activated EDTA alone or in association orthophosphoric acid were the most effective.

The worst performances shown by the protocol consisted in the use of the two solutions without activation, showing that the ultrasonic activation can significantly improve the efficiency of the post-space cleaning procedures.

The different dentin surface obtained with the various protocols is functional to the different methods of adhesion mandatory for post cementation.

If the technique requires the use of a total-etch adhesive, the use of an association of activated irrigants that determine a smearless layer surface is preferred.

When self-etch bonding is used, in which the adhesion interface is made by the smear layer, a less aggressive treatment of the post space is indicated. In this case the treatment with ultrasounds activated EDTA appears the best mode of irrigation.
